# Injected Drug Addiction-Associated Swollen Hands: A Case Report of Methylamphetamine-Related Unilateral Drug Addiction-Related Puffy Hand Syndrome

**DOI:** 10.7759/cureus.51545

**Published:** 2024-01-02

**Authors:** Philip R Cohen

**Affiliations:** 1 Dermatology, University of California, Davis Medical Center, Sacramento, USA; 2 Dermatology, Touro University California College of Osteopathic Medicine, Vallejo, USA

**Keywords:** unilateral, syndrome, swelling, puffy, methylamphetamine, hand, edema, drug, bilateral, addiction

## Abstract

Puffy hand syndrome occurs in addicts who have injected drugs either intravenously, intradermally, or subcutaneously. It usually presents as bilateral reversible pitting edema of the hands; less frequently, it occurs unilaterally. The forearms and arms may also be affected. The onset of puffy hand syndrome can occur while the patient is still injecting drugs; however, it can initially appear several years after injection of the drug has been discontinued. Infection with hepatitis C is a common comorbidity. A 47-year-old man is described who had a 20-year history of injecting methylamphetamine only into his non-dominant left arm, forearm, and hand and experienced his second episode of unilateral puffy hand syndrome four years after discontinuing injecting the drug and three years after his initial episode; he also had hepatitis C infection. He presented with erythema and pitting edema of his left hand and forearm. Cellulitis was initially suspected, and he was admitted to the hospital for intravenous antibiotics; all cultures were negative for pathogens. The erythema and swelling resolved after five days of therapy. Puffy hand syndrome has been associated with various drugs; it has also been observed to occur in women during pregnancy and occasionally associated with acrocyanosis. The diagnosis is often not originally entertained by the clinician; the condition is often initially treated empirically as an infection. Serologic evaluation is typically negative for rheumatologic diseases, such as systemic lupus erythematosus and scleroderma, and cultures of the skin and blood are usually negative for pathogens. Radiologic assessment (such as roentgenograms, ultrasound to rule out venous thrombosis, computed tomography, magnetic resonance imaging, venogram, and lymphangiogram) may be performed, to exclude other conditions. Skin biopsy of the affected edematous hand occasionally demonstrates granulomatous inflammation and foreign bodies (suggestive of starch or injection additives) in the dermis. The edema for some of the patients with puffy hand syndrome was successfully treated with daily bandaging with compression stockings. The pathogenesis of puffy hand syndrome is considered to be multifactorial: damage to the veins, injury to the lymphatic system, and direct toxicity of the injectable drugs to the vascular structures.

## Introduction

Puffy hand syndrome describes the erythematous and edematous presentation of one or both hands. Initially, the edema is pitting, and subsequently, the swelling is indurated. All affected individuals had a prior history of injectable drug use [1-20].

The essential feature of the syndrome, either a unilateral edematous hand or bilateral puffy hands, was originally described in New York City drug addicts [1]. Subsequently, puffy hand syndrome has been observed worldwide [6,7,9-11,15,16,18,19]. The syndrome has been observed in not only drug users following intravenous delivery of the drug but also individuals who inject the drug either intradermally or subcutaneously [1-20].

A man with a history of injecting methylamphetamine into his non-dominant arm presented with his second episode of swelling of his left arm, forearm, and hand. He was empirically treated with systemic antibiotics for a suspected infection; after all additional investigations were negative, the diagnosis of puffy hand syndrome was established. The features of puffy hand syndrome are reviewed; importantly, puffy hand syndrome can not only mimic a skin and soft tissue infection but also occasionally occur concurrent with bacterial infection [4,5,12,17].

## Case presentation

A 47-year-old right-handed man presented with new and rapid onset of swelling of his left hand which had begun five days earlier. His hand was red and painful to touch. He was not febrile.

He was a homosexual man. He was human immunodeficiency virus positive and had a chronic hepatitis C infection. His past medical history was significant for 20 years of injecting methylamphetamine.

He only injected the drug into his non-dominant left arm. Injection sites included the antecubital veins and the superficial veins of his forearm. In addition, he had also performed intradermal or subcutaneous injections not only on his distal left forearm but also on his left wrist and left hand.

He had discontinued injecting the drug four years earlier. Three years ago, he had been admitted to the hospital for presumptive cellulitis of his left arm, which had presented with similar symptoms to this current episode. He received treatment with intravenous antibiotics and the swelling resolved; all the cultures that had been performed for infectious organisms had been negative.

**Figure 1:**
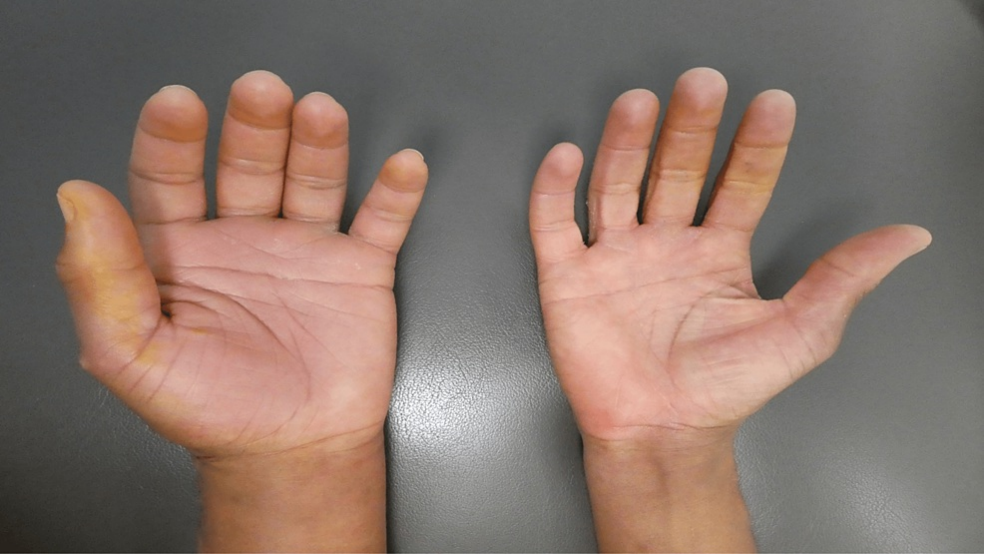
Puffy hand syndrome affecting the left hand The palmar surface of the left and right hands of a 47-year-old man with unilateral enlargement of his left hand.

Cutaneous examination showed dramatic swelling of both the dorsal and palmar aspects of his left hand. The pitting edema of his left hand was readily observed when his left hand was compared side-by-side to his right hand. In addition to the hand, the left thumb and all his fingers were swollen (Figure 1).

**Figure 2:**
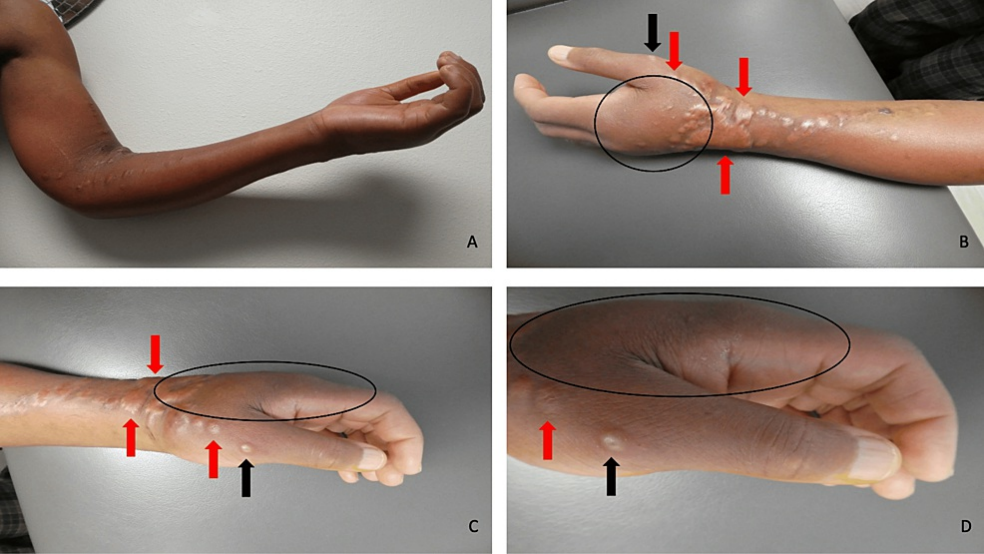
Drug addiction-associated puffy hand syndrome Views of the left arm (A), forearm (B and C), and hand (A, B, C, and D) demonstrate swelling that predominantly affects the dorsal left hand (circled by black oval). There are numerous keloidal scars on the forearm and hand (red arrows). A dried blister, which clinically mimicked a pustule, is noted at the base of the thumb (black arrow).

His left arm and forearms were not only swollen but also erythematous. He had numerous hypertrophic scars at prior sites of intravenous and intradermal or subcutaneous injections. The left hand was edematous. At the base of the thumb, a dried blister that morphologically mimicked a pustule was observed; the blister was deroofed and the culture performed was negative for bacteria (Figure 2).

He was admitted to the hospital. Serial blood cultures were all negative for pathogens. He received intravenous vancomycin and ceftriaxone. Ultrasound was negative for a deep venous thrombosis.

After five days of intravenous therapy, the left arm and forearm erythema had resolved. Also, the swelling of his left hand had decreased. He was discharged from the hospital with 10 days of doxycycline (100 milligrams twice daily) and cephalexin (500 milligrams four times daily). Correlation of his history of injectable drug use, his clinical presentation, the negative ultrasound for deep venous thrombosis, the negative cultures for pathogens, and the resolution of the erythematous edema of his left hand, forearm, and arm established a diagnosis of puffy hand syndrome.

## Discussion

Puffy hand syndrome refers to the swollen hands that may be observed in individuals with a prior history of injecting drugs into the affected upper extremity. Most of the addicts have performed proximal intravenous injections of the drugs into their arm. However, puffy hand syndrome has also been noted following either intradermal or subcutaneous injections into the arm, forearm, and/or hand [1-20].

A letter to the editor from Dr. Hans Abeles was published on November 18, 1965, in the correspondence section of the New England Journal of Medicine. He worked for the Departments of Correction and Health in the city of New York City which is in the state of New York (in the United States of America). He described the puffy hand sign of drug addiction in the addicts who typically injected the drug into the veins in the cubital regions of their arms [1].

Dr. Abeles noted that the puffy hands were usually bilateral and that there were changes to the contour of the dorsal aspect of the hands; since the hand was slightly edematous and had a smooth surface, the veins and tendons were no longer visible. He observed neither any pitting edema of the smooth thin skin of the dorsal hand nor any changes in the forearm skin. He attributed the pathogenesis of puffy hands to most probably be due to impaired circulation [1].

Subsequently, clinicians have also observed the edema to initially be pitting. They have also noted addicts in whom the syndrome not only involves the hand but also the forearm and arm. In addition, they have proposed that the etiology of puffy hand syndrome is multifactorial and that it can result from not only proximal intravenous injections but also proximal and/or distal intradermal and subcutaneous injections into the affected extremity [1-20].

Injected drug addiction-associated swollen hands (also referred to by the acronym InDadDash) was originally described as puffy hand sign by Dr. Abeles in 1965 (Table 1) [1-20]. Subsequently, descriptive nomenclature was used to designate the unique clinical presentation such as chronic edema of the hands and lymphedema [2,6]. Another group of investigators attempted to rename puffy hand syndrome to hepatitis-C hands to emphasize the often-associated undiagnosed hepatitis C infection in these individuals; however, this new designation did not gain acceptance in the medical literature [2].

**Table 1:** Nomenclature of InDadDash ^a^Chronic edema of the hands was introduced by Drs. Alexander W. Young Jr. and Fredrick R. Rosenberg in 1971 in a case report of a heroin user with a review of the cutaneous stigmas of heroin addiction [5]. ^b^Studdiford et al. considered undiagnosed hepatitis C infection to be associated with "puffy hand sign" [2]. In 2007, they introduced the term "hepatitis-C hands" to underscore the linkage of clinical presentation of the hands and the associated infection. Although several of the individuals, including the patient in this report, with puffy hand syndrome also had hepatitis C infection, the term did not gain acceptance in the medical literature [7,8,10,15,20]. ^c^The term "injected drug addiction-associated swollen hands" and its acronym (InDadDash) were introduced in this report to emphasize the pathogenesis of this condition to not only intravenous but also cutaneous and subcutaneous injection of the drug. The acronym InDadDash is derived as follows. "In" is from the first two letters "in" from the word injected. "Dad" is from the first letter "d" from the word drug and the first two letters "ad" from the word addiction. "Dash" is from the third letter "d" from the word addiction and the first letters "a", "s", and "h" from words associated, swollen, and hands. InDadDash: injected drug addiction-associated swollen hands

Nomenclature	References
Chronic edema of the hands^a^	[4,5]
Hepatitis-C hands^b^	[2]
Injected drug addiction-associated swollen hands^c^	Current report
Lymphedema	[6]
Puffy hand	[3]
Puffy hand sign	[1,2]
Puffy hand syndrome	[7-20]

Patients with puffy hand syndrome have been reported as case reports and studies in the medical literature (Table 2) [1-20]. Most of the reports only contain a description of only one or two patients. Six studies (Table 3) included more than two patients with puffy hand syndrome [3,4,6,10,18,19]. In addition, one letter commented that it was not uncommon to see puffy hand syndrome among the several hundreds of patients with intravenous cases of addiction that were seen each month [1].

**Table 2:** Number of patients with puffy hand syndrome included in publication ^a^There were six studies that each included more than two patients with puffy hand syndrome [3,4,6,10,18,19]. ^b^Dr. Abeles commented that several hundreds of cases of addiction were seen every month during the routine medical inspection of men admitted to the institutions of the Department of Correction in New York City. In addition, he also stated that it was not uncommon to see puffy hand syndrome in the addicts which was characterized by a definite change in the contour of the dorsal aspects of their hands [1].

Number of patients^a^	References
1	[2,5,8,11-17,20]
2	[7,9]
3	[18]
4	[3,6]
6	[4]
33	[10]
45	[19]
Several hundred^b^	[1]

**Table 3:** Studies involving patients with puffy hand syndrome PHS: puffy hand syndrome; Ref: reference

Year	Comment	Ref
1970	A senior registrar in the Skin Department at King's College Hospital in London evaluated the skin lesions in 54 drug addicts over a one-year period; each patient was seen at least once. The intravenous drugs included heroin and methadone or methadone alone (52 patients); nine included methylamphetamine and cocaine. PHS (described as lymphedema) was present in four patients.	[6]
1972	A study evaluating the pathogenesis of PHS involved many patients with PHS; however, only four patients were studied in-depth. Superficial cellulitis at an injection site required treatment in two patients; the opposite swollen hand was studied. The other two patients were admitted for hand surgery unrelated to their PHS. Evaluation included venograms (which was only able to be performed in two patients), lymphangiography (which was attempted in four patients and successfully performed in only one patient), and biopsies of the skin and subcutaneous tissues (in all four patients).	[3]
1974	Three physicians from the Division of Plastic Surgery of The Johns Hopkins Hospital and The Johns Hopkins University School of Medicine were evaluating drug injection injuries of the hands and forearms in addicts presenting to the emergency room during a period of four months. They provided a profile of 38 patients who were injecting either heroin (20 patients), cocaine (12 patients), methadone (three patients), or not specified (three patients). PHS (described as chronic edema and joint stiffness) was observed in six patients who injected the drugs subcutaneously: it was confined to the non-dominant hand in five patients and both hands in one patient who was ambidextrous. The other clinical manifestations of drug injection injuries included a painful nodule at the injection site (15 patients), edema, fever, and pain of acute sepsis (eight patients), a non-healing skin ulcer at the injection site (six patients), a cold, cyanotic hand from brachial artery injection (one patient), septic gangrene of a finger following an attempted digital vein injection (one patient), and a broken needle in the antecubital space (one patient).	[4]
2005	The diagnostic clinical features of 10 Indian patients (six women and four men) with pentazocine-induced ulcers were evaluated over a 12-month period. PHS was present in three women who had evidence of intravenous pentazocine abuse, but not in the other patient who were using other routes (predominantly intramuscular) to access the drug.	[18]
2006	French investigators performed a case-control study of the pathogenesis of PHS due to drug addiction. All the study participants were intravenous drug addicts (past heroin uses, mainly methadone-substituted) including 33 cases with puffy hands and 33 controls without puffy hands. Multivariant analysis demonstrated that significant risk factors for PHS were the following: women, injections in the hands, injections in the feet, and the absence of tourniquet use. However, high-dose sublingual buprenorphine misuse was not a significant PHS risk factor.	[10]
2022	In North India, the prevalence and pattern of dermatological manifestations among substance users across the Kashmir Valley was evaluated over an 18-month duration. PHS was observed in patients after long-term intravenous drug addiction in 45 (8.7%) of 515 injectable heroin users. The most common site was the non-dominant hand.	[19]

The incidence of puffy hand syndrome remains to be definitively established; however, it can be estimated based on four studies (Table 4) [4,6,18,19]. The studies ranged in size from 10 individuals to 515 patients. The incidence of puffy hand syndrome in these studies ranged from 7% to 30%; the median incidence of puffy hand syndrome was 12%. However, an incidence of 9% (58 patients with puffy hand syndrome of 617 patients studied) was calculated when the data from the individual studies was combined [4,6,18,19].

**Table 4:** Incidence of PHS PHS: puffy hand syndrome; Ref: references

Year	Number of patients with PHS	Number of patients in study	Percent of patients with PHS	Comment	Ref
1970	4	54	7.4	The skin lesions associated with the injection of narcotic drugs in 54 patients attending a treatment center in London, England, who were examined at least once over a period of 12 months. PHS was recorded as lymphedema.	[6]
1974	6	38	15.8	A profile of the clinical manifestations of drug injection hand injuries was conducted for 38 patients who presented with complaints regarding the hand or forearm during a four-month period to the emergency room at The Johns Hopkins University School of Medicine in Baltimore, Maryland, United States. Chronic non-pitting swelling (edema) of the hand and digits (and occasionally of the forearm as well) was seen in six of the patients.	[4]
2005	3	10	30	A study of 10 patients with pentazocine-induced ulcers seen in the Dermatology Outpatient Department of the All India Institute of Medical Sciences, New Delhi, India, between November 2000 and October 2002; three of the six women who had intravenous pentazocine abuse had PHS.	[18]
2022	45	515	8.7	A study to determine the prevalence and pattern of dermatological manifestations among substance users across the Kashmir Valley in North India for a period of 18 months from November 2018 to April 2020 identified 45 patients with PHS that developed post-chronic intravenous heroin use.	[19]
TOTAL	58	617	9.4	The incidence of PHS ranged from 7.4% to 30%; the median incidence of PHS was 12.25%.	[4,6,18,19]

Puffy hand syndrome has been associated with the injection of various psychotropic substances (Table 5) [1-20]. Heroin diluted in the antimalarial quinine or quinine by itself has also been injected [3,4]. Also, one person injected poppy seed tea [11].

**Table 5:** Injected drugs or substances in patients who develop puffy hand syndrome

Drug	Reference
Amphetamine	[11]
Buprenorphine	[7,8,16]
Cannabis	[11]
Cocaine	[4,6,7,9,10]
Glutethimide	[11]
Heroin	[3-7,9,10,12,19,20]
Heroin and quinine	[3]
Hydrocodone	[11]
Methadone	[4,6,9-11]
Methylamphetamine	[6,13,17]
Morphine	[8]
Not specified	[1,2,4,14,15]
Opiates	[13]
Pentazocine	[18]
Poppy seed tea	[11]
Quinine	[4]
Suboxone	[8]

Heroin was the most frequently associated injected drug. Other common drugs included cocaine, either amphetamine or methylamphetamine, methadone, and buprenorphine. One group of investigators determined that high-dose sublingual buprenorphine misuse was not a significant risk factor for puffy hand syndrome [10].

Several associated conditions have been observed in patients with puffy hand syndrome. Including the man in this report, hepatitis C infection was most common associated illness [7,8,10,15,20]. Indeed, one group of investigators suggested that the puffy hands be renamed hepatitis-C hands [2].

Puffy hand syndrome was observed in three women during their pregnancy (Table 6) [8,9,20]. All the women had their onset of puffy hand syndrome prior to the associated pregnancy [8,9,20]. One of the women also had acrocyanosis (without Raynaud's phenomenon) of both hands with concurrent palmar erythrosis and telangiectasias [9].

**Table 6:** Presentation of puffy hand syndrome in pregnant women ^a^As per the case report: "This edema appeared during pregnancy". A: age (years); Alc: alcoholic; BP1: bipolar 1 disorder; Bup: buprenorphine; BHCT: bilateral hand cellulitis and tenosynovitis; C: case; CG: compression gloves; CJP: chronic joint pain; CR: current report; CS: compression sleeves; F: female, G: gender, HCV: hepatitis C virus antibody positive (RNA negative); LTF: lost to follow-up; M: male; Morph: morphine; NOLW: non-healing open leg wounds; NS: not stated; PMH: past medical history; Ref: references; RNA: ribonucleic acid negative; SM: smoker; Sub: suboxone; T2: trimester 2 of pregnancy; wk: weeks' gestation

A	Onset	Injected drugs	PMH	Comment	Tx	Ref
27	NS^a^	Cocaine	Alc, Sm	From age 16 to 25 years, she used intravenous cocaine. She restarted the cocaine and nasal heroin for six months prior to presentation; she transiently took Bup. Bilateral hand and forearm edema initially appeared after five years of cocaine use; it also appeared during pregnancy. Initially, it was fluctuating and then permanent. She also had acrocyanosis (without Raynaud's phenomenon) of both hands, telangiectasia, and palmar erythrosis.	CS LTF	[9] C2
34	28 wk	Heroin	BHCT, HCV, NOLW	During T2, the patient (who had self-injected heroin into her hands and lower legs for years and was undergoing opioid dependence treatment with oral Bup) was referred from the obstetrics and gynecology service to the dermatology service for the evaluation of severe pitting and woody edema of all four limbs. Since age 32 years, she had a history of intermittent, disfiguring edema, erythema, and pruritus of the bilateral hands and lower legs. The only lab abnormality was an elevated (19 units) immunoglobulin M anti-beta-2-glycoprotein, which can be associated with pregnancy.	CG LTF	[20]
35	T2	Bup, heroin, Morph, Sub	BP1, CJP	From age 25 years, for four years, she injected the drugs intravenously. She stopped her drug use at age 29 years; three years later, at 32 years, she developed intermittent and painless, symmetric swelling of both hands. At 34 years, two years later, beginning during T2, persistent edema with new erythema of the hands and feet developed. The edema and erythema of her hands still had persisted at 11 months postpartum (for a total duration of 15 months) when she presented at age 35 years to the dermatology service for evaluation.	NS LTF	[8]

Acrocyanosis has been observed in at least three women with puffy hand syndrome (Table 7) [7,9,15]. Two of the women began using intravenous drugs as teenagers (beginning at 14 years or 16 years); both women were also smokers and alcoholics. The onset of puffy hand syndrome after initiation of intravenous drug use was not stated for one woman; it began after five years or 15 years of drug use in the other women. Two of the women had hepatitis C; the other woman's puffy hand syndrome presented during her pregnancy [7,9,15].

**Table 7:** Acrocyanosis in patients with PHS A: age (years); C: case; CR: current report; F: female; G: gender; PHS: puffy hand syndrome; Ref: references

A	G	Comments	Refs
27	F	The woman, a smoker and alcoholic, was seen in rheumatology consultation. She used intravenous cocaine from 16 to 25 years of age. Six months prior to presentation, she restarted intravenous cocaine and nasal heroin. At age 21 years, she developed bilateral PHS; the edema appeared during pregnancy. Initially, the edema fluctuated and then it became permanent. The clinicians observed acrocyanosis of both hands, telangiectasia, and palmar erythrosis; there was no Raynaud's phenomenon. Anti-nuclear antibodies were present at low titers (1/160) without specificity, there were no rheumatoid factor or anti-citrullinated peptide antibodies, and the complement and the protein electrophoresis were normal.	[9] C2
27	F	The woman had a history drug addiction that involved injection of narcotic drugs in the dorsal aspects of both hands that had ceased by age 24 years. From age 25 years, she developed bilateral PHS that presented with chronic swelling of both hands associated with recurrent digital erythema; all of her fingers had marked acrocyanosis of the dorsal fingertips and stable swelling and erythema with a few superficial telangiectasias. Rheumatologic evaluation was negative for antinuclear antibody titer, ribonucleoprotein, antitopoisomerase, centromere, Sjogren syndrome A, Sjogren syndrome B, and Smith antibody titers. Examination was negative for human immunodeficiency virus but positive for hepatitis C virus.	[15]
34	F	The woman was a smoker and an alcoholic. At age 14 years, she began to use intravenous cocaine and heroin, which was repeatedly replaced by buprenorphine. Her past medical history was also significant for hypothyroidism, poor dental condition, chronic hepatitis C (without cryoglobulin diagnosed at age 28 years), venous thrombosis, and infections (abscesses and erysipelas). PHS began at age 29 years, and it was predominantly on the right upper extremity; the edema increased in volume and extended from the hands and fingers to the forearms. At age 34 years, she was hospitalized for the treatment of the edema of her upper extremities. Cutaneous examination also showed acrocyanosis and excoriated lesions of the forearms. The edema improved after 11 days of daily multi-layered bandages; she was discharged with a compression stocking from her fingers to forearm; the volume of edema was stable with the wearing of the elastic compression and regular practice of self-bandaging at a follow-up visit 18 months later.	[7] C2

The onset of puffy hand syndrome is variable. Usually, it begins several years after the cessation of intravenous drug use [8]. However, it can appear either during drug use or shortly after discontinuation of drug addiction [9].

Initially, the edema is non-pitting and not affected by elevation of the arm [3,8,14,16]. Often, it will resolve spontaneously after the early episodes. Subsequently, the edema becomes non-pitting, indurated, and persistent [1-20].

Puffy hand syndrome is usually painless; however, uncommonly, the hands can be tender [7]. However, like the man in this report, the presentation may mimic a skin and soft tissue infection [12,17]. Indeed, some of the patients with puffy hand syndrome had a concurrent bacterial infection [4,5].

There is frequently associated erythema of the swollen hands. The edema most commonly affects the dorsal surface of the hands and fingers. Typically, the skin of the dorsal hand is thin and smooth with loss of the surface anatomy; the extensor tendons and dorsal veins of the hands are frequently not able to be observed [2,3].

Movement of the hands and fingers varies in patients with puffy hand syndrome. Some investigators noted that the function of the hands and digits were not impaired [3,10]. Other researchers found either that the patient had a limited range of motion of the hands or that the edema caused the person to have difficulties with gripping and dexterity [14].

Puffy hand syndrome is usually bilateral [1-4,7-10,12-20]. However, like the man in this report, unilateral presentation of puffy hand syndrome has been observed (Table 8) [1,4,7,11,13,17,19]. When the syndrome only affects one hand, the patient has typically used his dominant hand to inject the drug into the non-dominant hand, forearm, and/or arm [4].

**Table 8:** Features of patients with unilateral PHS A: age (years); Amp: amphetamine; Bup: buprenorphine; CR: current report; HCV: hepatitis C virus; Hdc: hydrocodone; Gt: glutethimide; LTF: lost to follow-up; Meth: methadone; Methyl: methylamphetamine; Morph: morphine; NS: not stated; PHS: puffy hand syndrome; PST: poppy seed tea; Ref: references

A	Injected drugs	Comment	Ref
34	Codeine, heroin	The woman had used intravenous heroin and cocaine for 20 years, in addition to buprenorphine. PHS began after 15 years of drug use, at age 29 years; bilateral edema, predominantly on the right side, involved her fingers, hands, and forearms. Other complications included acrocyanosis, chronic hepatitis C, venous thrombosis, abscesses, and erysipelas. Multi-layer bandages were applied daily in the hospital for 11 days, and the volume of the upper extremities significant decreased. She received a custom-made compression stocking which extended from her proximal fingers to the forearm when she was discharged from the hospital. She continued to regularly perform self-bandaging and to wear the elastic compression stocking; examination, at 18 months of follow-up, showed that the improvement of her edema was stable.	[7] C2
37	Opiates, Methyl	The man had used intravenous opiates and Methyl until age 35 years. From age 35 to 37 years (for two years), he had sobriety of his drug use; at age 37 years, one month prior to presentation, he restarted using intravenous opiates and Methyl. At age 36 years, eight months prior to presentation, he began to develop swelling of his fingers and hands. Initially, the symptoms were intermittent; however, in the last four months, they became persistent, and he had only had a limited range of motion in his fingers and wrists, with loss of hand dexterity and difficulty picking up objects or putting on socks and shoes. Importantly, the swelling stated only in the right (non-dominant) hand; yet, within a few weeks, the swelling also affected his left hand. The patient decided to quit intravenous drug use and undergo drug rehabilitation and psychiatric counseling. At follow-up (duration not provided), he had not regained much movement in his hands and fingers.	[13]
47	Methyl	The man had used intravenous Methyl since age 23 years and stopped using the drug at age 43 years. He was right-handed and only injected methyl into his non-dominant left arm; the injections were intravenous, intradermal, and subcutaneous into the arm, forearm, and hand. One year after stopping drug use, he developed swelling of his left arm; cultures for infectious organisms were negative, and the swelling resolved after intravenous antibiotics. Three years later, at 47 years, he again developed swelling of his left hand, forearm, and arm; again, the cultures for infectious organisms were negative, and the swelling resolved after intravenous antibiotics.	CR
49	Amp, cannabis, Hdc, Gt, Meth, PST	The woman, who otherwise appeared healthy, had non-pitting edema and erythema on her right hand and left foot for two years since age 47 years. However, since age 22 years, she had been injecting psychoactive drugs and other substances intravenously and subcutaneously. The injection locations included the cubital region, hands, and feet; however, her symptoms were predominantly unilateral only the right upper extremity and left lower extremity.	[11]
59	Methyl	The man had a history of intravenous drug use (Methyl); in addition, his urine toxicology was positive for Amp. He was afebrile and presented with unilateral swelling of the right hand, which was red, warm, and painful of three days' duration. Exam was suggestive of an acute skin and soft tissue infection: non-pitting edema, warmth, blanchable erythema, and decreased range of motion of the right hand. He was empirically treated with an intravenous antibiotic (vancomycin); there was not improvement after 48 hours. Laboratory studies (C-reactive protein, leukocyte count, and blood cultures), ultrasound, roentgenograms, and magnetic resonance imaging were all negative. A diagnosis of PHS was made. Within another 48 hours after adding upper extremity elevation in a Murphy sling, his right upper extremity edema, erythema, and pain improved and completely resolved after 96 hours.	[17]
NS	NS	Several hundred men with addiction were observed by Dr. Hans Abeles at the Department of Correction in New York City every month. Puffy hand sign was not an uncommon change to observed in the contour of the dorsal aspect of the hand in the addicts. He implied that PHS occurred unilaterally when he stated that the puffy hands were usually found bilaterally.	[1]
NS	Cocaine and heroin, Meth, NS	Chronic non-pitting swelling of the hand and digits (and occasionally also the forearm) and joint stiffness (PHS) was observed in six of 38 (16%) addicts with injuries of the hands and forearms seen during a four-month period at the emergency room of The Johns Hopkins Hospital. In five patients (all of whom were long-term addicts, ranging from eight to 22 years), the swelling was confined to only the non-dominant hand; it occurred in both hands in the sixth patient who was ambidextrous. Both active and passive finger mobility was impaired. At least one patient had concurrent bacteria culture-positive infections (from the flexor tendon sheath and forearm abscesses) with *Streptococcus* and *Staphylococcus*. All the patients were unable to locate their veins routinely injected the drugs subcutaneously. The injected drug was not specified (one patient), cocaine and heroin (three patients), or Meth (two patients).	[4]
NS	Heroin	In a study of 515 heroin users in North India, 8.7% (45 patients) had PHS. Non-pitting edema with a puffy appearance, especially involving the non-dominant hand, affected the dorsal side of the fingers and hand. The investigators observed that the condition developed after long-term intravenous drug addiction in whom tourniquet use while injecting the heroin was lacking. They also noted that PHS predominantly occurred in men and was most commonly located on the non-dominant hand followed by the dominant hand.	[19]

The diagnosis of puffy hand syndrome is made by excluding other causes of acute hand swelling. The rapid onset of a swollen hand can be a clinical sign of an infection of the skin and/or the underlying soft tissue. Therefore, a bacterial infection, such as cellulitis, is usually suspected, and systemic antibiotic treatment is often appropriately empirically initiated, like the reported patient [12,17].

The concurrent presentation of bilateral cellulitis of the hands is uncommon. Recurrent, painless edema, absence of fever, a normal leukocyte count, and subsequent negative skin and blood cultures for pathogens favor the diagnosis of puffy hand syndrome instead of cellulitis. However, when the hand edema is unilateral and the hand is tender, the possibility of cellulitis cannot be excluded. In addition to only one hand being affected by painful edema, fever and neutrophilia would favor the diagnosis of cellulitis; positive cultures of skin and/or blood for pathogens would also support the diagnosis of infection. Indeed, the diagnoses of puffy hand syndrome and infection (such as cellulitis or osteomyelitis) can occur simultaneously [4,5].

Although the diagnosis of puffy hand syndrome is usually not initially entertained by the clinician assessing a patient with either the new-onset or a recurrent episode of swollen hands, the possibility of this condition is more seriously considered once the evaluation for bacterial infection is negative and laboratory tests for other conditions, such as systemic lupus erythematosus and scleroderma, are negative [9,13-16,20]. Serologies for hepatitis C may concurrently be positive like the man in this paper [7,8,10,15,20]. The appropriate clinical history must be confirmed; the use of injected drugs may have occurred several years earlier, and neither the patient nor the clinician may be initially considering this relevant past medical history.

The clinical differential diagnosis of puffy hand syndrome is listed in Table 9 [2,3,8,9,12-14,17]. The diagnostic evaluation of some of the individuals with puffy hand syndrome may have included one or more radiological examinations [3-5,7,9,11-14,17]. Skin biopsy of the hand was also performed in some of the patients [3,8,11,14,20].

**Table 9:** Clinical differential diagnosis of puffy hand syndrome ^a^Complex regional pain syndrome, especially in its inflammatory phase, is in the clinical differential diagnosis [9]. ^b^These conditions can be distinguished by their systemic manifestations and characteristic pitting edema [2]. ^c^These infections usually resolved within a week, when treated with rest, soaks, and systemic antibiotics [3]. ^d^These infections are identified by tenderness, local heat, and local edema in the palm despite the more prominent dorsal swelling; the infections require formal incision and drainage in the operating room [3]. ^e^These include the following conditions: Meige's disease and Milroy's disease [9]. ^f^This is particularly in the early edematous phase of systemic sclerosis [8]. RS3PE: remitting seronegative symmetrical synovitis with pitting edema; CREST: calcinosis, Raynaud's phenomenon, esophageal dysmotility, sclerodactyly, telangiectasia

Diagnosis	References
Allergic contact dermatitis	[8]
Anasarca	[13]
Angioneurotic edema	[9]
Arterial thrombosis	[17]
Chondrocalcinosis	[9]
Complex regional pain syndrome^a^	[9,13]
Cirrhosis^b^	[2,9,13]
Congestive heart failure^b^	[2,8,9,12,13]
Erythromelalgia	[8,12,14]
Gout	[9]
Hypoalbuminemia (severe)	[13]
Infections	[2,3,9,13,17]
Bacterial	[9]
Cellulitis^c^	[2,9,13]
Deep palmar space infection	[13]
Fascial space infection on the hand^d^	[3]
Mycobacterial	[9]
Osteomyelitis	[17]
Parasitic (filariasis)	[9]
Skin and soft tissue infections	[17]
Viral (hepatitis C)	[9]
Irritant contact dermatitis	[8]
Liver failure	[8,12]
Lymphangitis	[12]
Lymphatic obstruction	[2,9]
Congenital hypoplasia of the lymphatic network^e^	[9]
Neoplastic disease-associated compression	[9]
Sentinel node removal	[9]
Vessel harvesting for coronary bypass graft surgery^b^	[2]
Vessel harvesting for vascular bypass graft surgery^b^	[2]
Lymphedema	[13]
Axillary lymph node removal-associated	[13]
Irradiation-associated	[13]
Nephrotic syndrome^b^	[2,9,13]
Polymyalgia rheumatica	[9]
RS3PE	[9]
Renal insufficiency	[8,12]
Rheumatoid arthritis	[8,12,14]
Scleroderma	[8,9,12,14]
CREST syndrome	[9]
Systemic^f^	[8,9,12,14]
Sjogren syndrome	[9]
Systemic lupus erythematosus	[8,12]
Trauma	[9,17]
Carpal bone fractures	[9]
Nonspecific traumatic event to the dorsal hand	[17]
Metacarpal bone fractures	[9]
Venous obstruction^b^	[2,9,12,13,17]
Deep vein thrombosis	[9,12,13,17]
Superficial vein obstruction	[9,12,13]

Several diagnostic radiological tests have been performed in patients with puffy hand syndrome. These include radiographs of the hands (Table 10) [5,9,13,14,17]. Roentgenograms of the affected hand were negative for pathology in four of the patients with puffy hand syndrome [9,14,17]. However, the roentgenogram of the right hand of a 27-year-old woman with puffy hand syndrome showed diffuse soft tissue swelling. In addition, roentgenograms of both hands of a 24-year-old man with puffy hand syndrome showed abnormalities; several fingers of the left hand demonstrated osteomyelitis, multiple fingers on the right had showed sort tissue necrosis and septic involvement of the joints, and the fifth fingers on both hands showed periosteal changes [5].

**Table 10:** Radiographs of puffy swollen hands A: age (years); C: case; F: female; G: gender; M: male; Ref: references

A	G	Comment	Ref
24	M	Roentgenograms of the hands showed osteomyelitis of the second, third, and fourth fingers of the left hand, soft tissue necrosis of the right second and fourth fingers, and probable septic involvement of the joints. There were periosteal changes in the fifth fingers of both hands.	[5]
27	F	Hand roentgenograms were normal; no arthritis.	[9] C2
37	M	Roentgenogram of the right hand showed diffuse soft tissue swelling.	[13]
40	M	Hand roentgenograms were normal; no arthritis.	[9] C1
40’s	F	Roentgenogram of the left hand showed neither erosions nor articular abnormalities.	[14]
59	M	Roentgenograms showed no fracture, foreign bodies, or gas formation.	[17]

Ultrasound of the reported patient's arm and of the extremity of other individuals with puffy hand syndrome has also been performed (Table 11) [7,9,12,17]. Including the reported patient with puffy hand syndrome, the ultrasound did not reveal a deep vein thrombosis in four patients who were evaluated [7,9,17]. In a 27-year-old man with puffy hand syndrome, the ultrasound showed an occlusive cephalic vein thrombosis in the right upper extremity [12]. In another patient with puffy hand syndrome, a 40-year-old man, the ultrasound only demonstrated subcutaneous edema [9].

**Table 11:** Ultrasound of patients with puffy swollen hands A: age (years); C: case; CR: current report; F: female, G: gender; M: male; Ref: references

A	G	Comment	Ref
27	F	The patient had been admitted in rheumatology consultation for bilateral hand edema. Musculoskeletal ultrasound was normal (no arthritis and no tenosynovitis). Ultrasound study of upper limb vessels was normal too.	[9] C2
27	M	The patient presented to an urgent care center with erythema and edema in his extremities and a small abscess with surrounding cellulitis on his right hand. Ultrasound showed occlusive cephalic vein thrombosis in the right upper extremity, without any evidence of thrombosis in the lower extremities.	[12]
40	M	The patient used corticosteroids (betamethasone) for 15 years, which had been prescribed by his family doctor, for edema of the two hands. Musculoskeletal ultrasound showed no arthritis, no synovitis, and no tenosynovitis; it only demonstrated subcutaneous edema.	[9] C1
40	M	The patient was hospitalized for the treatment of edema of his hands and forearms; he had a history of two episodes of superficial venous thrombosis of the upper limbs. Venous Doppler ultrasound of the upper limbs found no superficial veins, no deep radial and ulnar veins, and no sequelae of venous thrombosis.	[7] C1
47	M	The patient was hospitalized for edema of the left arm, forearm, and hand. The ultrasound was negative for a deep venous thrombosis.	CR
59	M	The patient presented with three days of right-hand swelling (non-pitting edema), redness, warmth, and pain. An ultrasound study did not reveal any deep vein thrombosis.	[17]

Venogram (Table 12) to evaluate for an associated venous thrombosis of the extremity has been conducted in two patients and attempted in two additional patients [3,13,17]. A study of the pathogenesis of the puffy hand of drug addiction focused on four individuals from many patients; dye was injected into a dorsal vein near the metacarpophalangeal joints. In two of the patients (who had extensive thrombosis of their hand veins), no venous flow could be demonstrated; the investigators assumed that the obstruction was located in the area of hand edema, and no attempt was made to identify the level of obstruction by performing more proximal injections. In the other two patients, the entire extremity was able to be evaluated; a normal venous pattern, as far proximal as the axilla, was observed [3].

**Table 12:** Computed tomography, magnetic resonance imaging, and venogram of patients with puffy swollen hands Ref: references

Test	Comment	Ref
Computed tomography	A 37-year-old man presented with intermittent swelling of his fingers and hands beginning eight months prior. Diffuse soft tissue edema and skin thickening, most prominent over the dorsal side, were observed on the computed tomography of his right hand. There were no bony cortical destruction and no enhancing fluid collection or gas in the soft tissues.	[13]
Magnetic resonance imaging	A 59-year-old man presented with three days of right-hand swelling (non-pitting edema), redness, warmth, and pain. A magnetic resonance imaging T2-weighted axial study of the right hand demonstrated diffuse myositis and edema of the intrinsic hand muscles; there were sparing of the tendons and no abnormal marrow changes. In addition, the T2-weighted magnetic resonance imaging study of the forearm demonstrated edema of the skin and subcutaneous tissue of the forearm.	[17]
Venogram	A study consisting of only four individuals, from a group of many patients with puffy hands, was performed. Two of the patients had extensive thrombosis of the veins of the hands, and venograms were not able to be successfully performed in these individuals. In the other two patients, venograms were successfully performed and showed a normal venous pattern in the entire extremity as far proximally as the axilla.	[3]

Lymphangiography has been attempted in several patients with puffy hand syndrome; however, in some of these individuals, the test was not able to be successfully performed (Table 13) [3,4,7,11]. Lymphoscintigraphy showed a normal lymphatic system in two women who were evaluated [7,11]. Abnormal findings were described in two patients: either bilateral lymphostasis and the absence of functional lymph nodes or a pattern consistent with deep lymphatic destruction [3,7]. In five of the patients in whom lymphangiography was attempted, the examination was unsuccessful: either the blue dye failed to stain the lymphatics (three patients), or none of the superficial lymphatics would allow cannulation (two patients) [3,4].

**Table 13:** Lymphangiography of patients with puffy swollen hands ^a^In a normal lymphangiogram, collateralization is not present, and the deep lymphatics are outlined rather than the superficial ones. ^b^Lymphatic obstruction, subsequent fibrosis, and lymphedema result after the injection of a quinine solution intralymphatically in animals. The frequent use of quinine to dilute heroin may be relevant to the damage of the patient's lymphatics. A: age (years); C: case; CR: current report; F: female; G: gender; M: male; NS: not stated; Ref: references

A	G	Comment	Ref
34	F	The patient had a history of recurrent venous thrombosis and infections; she had a five-year duration of edema of the upper limbs involving not only the forearms but also the hands and fingers. Lymphoscintigraphy did not show any abnormality of the axillary lymph node fixation.	[7] C2
40	M	The patient had edema of his hands and forearms and two prior episodes of superficial venous thrombosis of the upper limbs. Lymphoscintigraphy of the upper limbs showed bilateral lymphostasis with a few deep axillary lymph nodes and the absence of functional lymph nodes on the left side.	[7] C1
49	F	The patient had non-pitting edema and erythema on her right hand and left foot for two years. Lymphoscintigraphy of her upper and lower extremities showed a normal lymphatic system.	[11]
NS	NS	A study in which only four individuals, from a group of a large number of patients with puffy hands, were evaluated in-depth. Lymphangiography was not successful in three of the patients since the patient blue dye failed to stain their lymphatics. It was successfully performed in one patient whose hands had only recently started to swell; the pattern observed was consistent with deep lymphatic destructions. Specifically, a few superficial lymphatic channels along the radial aspect of the forearm and wrist were observed; extensive abnormal collateral channels around the elbow were visualized.^a^	[3]
NS	NS	A study evaluated 38 patients with drug injection injuries of the hands and forearms; six patients who injected the drugs subcutaneously had puffy hand syndrome (described as chronic edema and joint stiffness). Failure to demonstrate any superficial lymphatics that would allow cannulation resulted in abandoning the attempt to do lymphangiograms in two of the six patients.^b^	[4]

Computed tomography and magnetic resonance imaging were used in the evaluation of a patient with puffy hand syndrome (Table 12) [3,13,17]. Computed tomography of the edematous hand of a man with intermittent puffy hand syndrome showed diffuse soft tissue edema and skin thickening; these changes were most prominent over the dorsal side of the hand [13]. A magnetic resonance imaging study of the right hand of a man whose puffy hand syndrome presented with three days of non-pitting swelling of his right hand demonstrated diffuse myositis and edema of the intrinsic hand muscles and edema of the skin and subcutaneous tissue of the forearm [17].

A skin biopsy has been used in the evaluation of some of the patients with puffy hand syndrome (Table 14) [3,8,11,14,20]. The biopsy location was usually the dorsal hand; one woman had the skin of her dorsal index finger evaluated. The results of the skin biopsy were nonspecific or unremarkable in two women [8,14]. Two of the women had granulomatous inflammation and foreign body (either suggestive of starch or injection additives) in the dermis [11,20]. Four other individuals had dermal fibrosis and collagenization in the underlying fat [3].

**Table 14:** Biopsies of puffy hand syndrome ^a^Her past medical history included years of heroin self-injections to the hands and lower legs. ^b^She had a four-year history of injecting heroin, buprenorphine, suboxone, and morphine into the dorsal side of both hands. Three years after stopping intravenous drug use, she developed hand swelling for the prior 6.25 years. ^c^She had a remote history of intravenous substance abuse. ^d^She had used psychoactive drugs and other substances from the age of 22 years; more recently, she was using powdered methadone tablets intravenously and subcutaneously. Initially, she self-injected the drugs into the cubital region. ^e^Four patients (whose age and gender were not described) admitted to long-standing use of the dorsal veins of the hands for intravenous injections. The duration of swelling was not reported. All four patients were biopsied. A: age (years); Dur: duration of puffy hands in years; F: female; G: gender; NS: not stated; Ref: references

A	G	Dur	Location	Microscopic evaluation	Ref
34	F	2^a^	Left dorsal hand	Perivascular lymphocytic and neutrophilic infiltrate, small vessel engorgement, and dermal necrosis; also small foci of granulomatous inflammation with polarizable, refractile foreign material in a Maltese-cross pattern highly suggestive of starch. The latter findings were consistent with a cutaneous foreign body granuloma containing starch.	[20]
35	F	6.25^b^	Dorsal slide of her hands	Small vessel ectasia and minimal perivascular lymphocytic infiltrate. The results were interpreted as nonspecific findings.	[8]
40s	F	10^c^	Left dorsal index finger	Sparse perivascular lymphocytic infiltrate with focal spongiosis, rare dyskeratosis, and telangiectasias. Neither vacuolar interface changes nor closely packed, hyalinized collagen bundles were appreciated. Colloidal iron stain highlighted trace interstitial mucin. The biopsy results were interpreted to be relatively unremarkable.	[14]
49	F	2^d^	Right hand	Slightly sclerotic dermis with dilated blood vessels, lymphatics predominantly in the superficial dermis, and a sparse perivascular lymphocytic infiltrate. Some multinucleated giant cells in the deep dermis contained birefringent crystal-like foreign body particles, resembling foreign bodies from the additives of injections.	[11]
NS^e^	NS^e^	NS^e^	Dorsum of the hand	Biopsies of the skin and subcutaneous tissue from all four patients were similar. There was extensive dermal fibrosis and collagenization in the subcutaneous tissue.	[3]

The treatment of puffy hand syndrome depends on the manifestations of the patient (Table 15) [7-9,14,17]. For those individuals in whom the syndrome is initially presenting, the edema may spontaneously resolve once the episode has completed. Similar to the man in this report, resolution of both his primary episode and his recurrent episode occurred after completion of systemic antibiotics; however, laboratory studies did not demonstrate an infection. A 59-year-old man whose initial episode of puffy hand syndrome mimicked a skin and soft tissue infection demonstrated no improvement with systemic antibiotics; however, all manifestations of edema spontaneously and completely resolved with elevation of the affected right hand and forearm [17].

**Table 15:** Treatment of patients with PHS A: age (years); C: case; CR: current report; F: female; G: gender; M: male; PHS: puffy hand syndrome; Ref: references

A	G	Comment	Ref
27	F	She used intravenous cocaine from age 16 to 25 years; six months prior to presentation, she restarted intravenous cocaine and nasal heroin; she transiently took buprenorphine. At age 21 years, bilateral PHS appeared during pregnancy. The clinicians prescribed compression sleeves, but did not know if they improved her edema.	[9] C2
34	F	She had used intravenous heroin and cocaine for 20 years, since age 14 years, in addition to buprenorphine. She had chronic hepatitis C. At age 29 years, five years prior, bilateral PHS began; it predominantly affected her fingers, hand, and forearm on the right side. Other complications included acrocyanosis, venous thrombosis, abscesses, and erysipelas. During hospitalization, daily multi-layer bandages were applied for 11 days with significant decrease in the volume of the upper extremities. At discharge, a compression stocking from the proximal fingers to the forearm was custom-made. Wearing of the elastic compression stocking and the regular practice of self-bandaging resulted in stable improvement of the edema at follow-up 18 months later.	[7] C2
35	F	She had injected buprenorphine, heroin, morphine, and suboxone intravenously for four years, beginning at age 25 years. At age 29 years, she stopped her drug use. However, three years later, at age 32 years, she developed intermittent and painless, symmetric swelling of both hands. Subsequently, two years later, beginning during her second trimester of pregnancy, at 34 years old, persistent edema with new erythema of the hands and feet developed. The edema and erythema of her hands still had persisted at 11 months postpartum (for a total duration of 15 months). Treatment with compression gloves was recommended, and she was referred to a lymphedema clinic. Response to treatment was not described.	[8]
40	M	He used heroin intravenously from age 17 to 23 years; he then substituted methadone. At age 25 years, he developed bilateral PHS; the edema persisted and remained stable over time. At age 40, 15 years later, treatment with compression sleeves was initiated; during the subsequent three months, the patient noted an improvement of the hand's edema.	[9] C1
40	M	He used intravenous heroin from age 26 to 35 years; it was replaced by buprenorphine. He also had hepatitis C which had been treated (with recombinant interferon alpha-2b and ribavirin) and superficial venous thrombosis of both arms. Bilateral PHS began at age 30 years; initially, the edema was intermittent and then became permanent. At age 40 years, he was hospitalized to treat the edema of the hands and forearms; multi-chamber pneumatic pressure therapy had been unsuccessful. His daily treatment consisted of multi-layered bandages consisting of elastic bands on foam padding for 11 days. Not only the volume of the edema decreased, but also the diameter of the hand decreased. An elastic compression stocking was given at the time of discharge. After 10 months, he again required hospitalization because of noncompliance and an increase in the edema volume; this again resulted in a reduction of the edema volume.	[7] C1
47	M	The man had injected methylamphetamine either intravenously, intradermally, or subcutaneously into his non-dominant left arm, forearm, and hand for 20 years from age 23 to 43 years. He developed unilateral PHS one year after stopping drug use; at 44 years of age, there was initial swelling of his left arm; cultures for infectious organisms were negative and the swelling resolved after intravenous antibiotics. Three years later, at 47 years, he again developed swelling of his left hand, forearm, and arm; like the initial episode, the cultures for infectious organisms were negative. The swelling resolved spontaneously after intravenous antibiotics.	CR
40’s	F	The woman had a remote history of intravenous substance abuse. She presented with a 10-year history of bilateral PHS, untreated hepatitis C infection, and polycythemia vera. She was referred for repeated sessions of manual lymphedema decongestion and occupational therapy; also, treatment of her hepatitis was recommended. The results of therapy were not described.	[14]
59	M	He had a history of intravenous methylamphetamine use; he claimed to have stopped injecting the drug and he had received buprenorphine treatment; however, his urine toxicology was positive for amphetamine. His initial episode of PHS mimicked an acute skin and soft tissue infection. The edema showed no improvement after 48 hours of empiric systemic antibiotics; upper extremity elevation in a Murphy sling was initiated. The edema, erythema, and pain improved after 48 hours of this treatment and completely resolved after 96 hours.	[17]

Some of the patients with recurrent and persistent edema caused by puffy hand syndrome were treated with conventional approaches that are used to treat lymphedema from other etiologies (Table 15) [7-9,14,17]. Compression gloves for the hands and stockings for the forearms were found to be helpful in decreasing the volume of the edema and to decreasing the diameter of the swollen hands. Chronic treatment was noted to be necessary to maintain improvement; a lapse in therapy was associated with edema recurrence [7].

The mainstay of treatment of persistent edema associated with puffy hand syndrome is compression therapy for lymphedema. In addition, prevention of infection is paramount. Also, it is important to provide protection from cold to prevent acrocyanosis [9].

French investigators, in 2006, performed a case-control study of the pathogenesis of puffy hand syndrome due to drug addiction. All the study participants, 33 patients with puffy hands and 33 control patients without puffy hands, were intravenous drug addicts (who mostly consisted of past heroin users who were currently being treated with methadone); the mean age of the participants was 34 years. Multivariant analysis was performed; significant factors for puffy hand syndrome included the following: sex (women), injections in the hands, injections in the feet, and the absence of tourniquet use. The researchers had suspected high-dose sublingual buprenorphine misuse to influence the development of puffy hand syndrome; however, their analysis did not show this variable to be a significant risk factor for the development of puffy hand syndrome [10].

The pathogenesis of puffy hand syndrome is favored to be multifactorial; the edema develops whether the injection is intravenous (in the cubital veins or in the dorsal hand veins), or subcutaneous, or intradermal. The etiology of puffy hand syndrome is related to the pathologic changes that occur in the venous system, the pathologic changes that develop in the lymphatic system, and the effect from the toxicity of the injected drugs. Transient swelling eventually becomes permanent; indeed, the puffy hand persists even after the addict stops injecting the drug [2,3,7-12,14,16].

Alteration of the lymphatics has been suggested to have a causative role in the development of puffy hand syndrome. Puffy hand syndrome occurs not only from lymphatic insufficiency but also as a consequence from lymphatic obstruction. In addition, puffy hand syndrome results from superimposed local scarring secondary to inflammatory reactions at injection sites and the destruction of lymphatics and venules secondary to septic microthrombi caused by poor aseptic technique [1,7,8].

Puffy hand syndrome, in part, occurs secondary to the direct toxicity of the drugs injected; the local vasculature is weakened by the chronic destruction of the drug [8]. Buprenorphine and heroin can be directly toxic to the vessels; also, accompanying contaminants within the drug, such as talc and flour, can cause vessel destruction [8,9]. For example, injection of quinine destroys lymphatics; therefore, repeated insults from heroin diluted with quinine can result in vessel damage and puffy hand syndrome [3].

## Conclusions

Intravenous, intradermal, or subcutaneous injection of drugs can result in puffy hand syndrome. Frequently, puffy hand syndrome initially appears several years after injection of the drug has been discontinued. Not only the hands but also the forearms and arms can be affected. It usually presents as bilateral reversible pitting edema; subsequently, the edema becomes persistent and non-pitting. A 47-year-old man is reported with unilateral puffy hand syndrome; he began injecting methylamphetamine at age 27 years only into his non-dominant left arm, forearm, and hand. He stopped drug use at age 43 years; one year later, he developed his initial episode of puffy hand syndrome, and three years thereafter, he experienced his second episode. He presented with left hand and forearm erythema and pitting edema. Cellulitis was initially suspected; however, all cultures were negative for pathogens. The erythema and swelling resolved after five days of intravenous antibiotics. Similar to the reported patient, infection with hepatitis C is a common comorbidity in individuals with puffy hand syndrome. Daily bandaging with compression stockings may be helpful to decrease the edema. Damage to the veins, injury to the lymphatic system, and toxicity of the injectable drugs to the vascular structures are postulated to contribute to the pathogenesis of puffy hand syndrome. The diagnosis of puffy hand syndrome is often not considered by the clinician until repetitive episodes of erythematous edematous swelling of the hands and upper extremity have been observed; in particular, the cultures of the skin and blood are typically negative for pathogens, and the serologic evaluation is negative for systemic lupus erythematosus and scleroderma. However, similar to the reported patient, puffy hand syndrome is frequently treated empirically as an infection before the correct diagnosis is determined.
